# What do we know about pediatric renal microlithiasis?

**DOI:** 10.15171/jrip.2017.13

**Published:** 2016-11-14

**Authors:** Mohammad Amin Fallahzadeh, Jafar Hassanzadeh, Mohammad Hossein Fallahzadeh

**Affiliations:** ^1^Shiraz Nephrourology Research Center, Shiraz University of Medical Sciences, Shiraz, Iran; ^2^Department of Biostatistics and Epidemiology, Shiraz University of Medical Sciences, Shiraz, Iran

**Keywords:** Renal Microlithiasis, Renal Stone, Urolithiasis, Nephrolithiasis, Pediatrics, Child

## Abstract

Renal or calyceal microlithiasis is a common disorder with increasing prevalence especially in infants and younger children. The main presenting symptoms and the underlying metabolic abnormalities of renal microlithiasis are similar to renal stone. Although renal microlithiasis is considered as a main problem of the health system with diverse etiologies, our information about its natural course is very limited. Hence, further investigations to make an appropriate clinical approach to this entity is mandatory. Also, general practitioners, pediatricians, nephrologists and urologists have to be well educated regarding renal microlithiasis for early diagnosis, appropriate evaluation and proper management of this entity. In this review study, we focused on collection of the present information about different aspects of renal microlithiasis in children

**Implication for health policy/practice/research/medical education:**

Pediatric renal microlithiasis is a common entity which can present with non-specific symptoms in infants and young children. With technological advancements and growing expertise, renal microlithiasis can be diagnosed more easily than before. Better understanding of different aspects of this disorder discussed in this article will result in early diagnosis and appropriate management of renal microlithiasis. This, in turn, will decrease the prevalence of renal stone.

## Introduction

Nephrolithiasis in infancy and childhood is substantially different from adult population in many aspects, including the epidemiology, etiology, symptoms, signs, imaging modalities and management (1). Renal stone in children should not be underestimated because of associated significant morbidity and higher recurrence rate as compared to adults (2).

Since metabolic causes are expected in most infants and children with renal stone, diagnostic evaluation is mandatory for every pediatric patient with the first documented renal stone to find the underlying metabolic disorders that can lead to recurrent urolithiasis (3). Also, renal stone in infancy and childhood is usually a multifactorial disorder related to some other underlying diseases and conditions including heredity, ethnicity, climate and nutritional habits (4).

Renal microlithiasis or microcalculi (RM) is defined as ultrasonographic detection of hyperechogenic deposits smaller than 3 mm in diameter in renal calyces, pelvis or ureter. If the microcalculi are located only in the calyces, they are called renal calyceal microlithiasis (5-8). There are only few reports on diagnosis, investigation, management, long term follow-up and final outcome of RM. Lack of controlled studies has hindered development of acceptable guidelines on investigation, management and follow-up of pediatric RM. Therefore, this review study was carried out to examine the clinical presentation and importance, metabolic predisposing factors and prognosis of RM during infancy and childhood.

## Materials and Methods

In this review study, we searched MEDLINE, EMBASE, the Cochrane Central Register of Controlled Trials (The Cochrane Library) to identify the relevant articles. Additionally, guideline websites, relevant nephrology meeting proceedings and reference lists of the related articles up to 30th of October 2015 were reviewed. The key words were renal microlithiasis, renal calyceal microlithiasis, renal microcalculi, renal stone, renal calculi, urolithiasis, nephrolithiasis, pediatrics, child and infant.

## Epidemiology

Urolithiasis can be discovered in all pediatric age groups (9). As compared to adults, renal stone is most probably less frequent in children and is more often associated with an underlying metabolic disorder (9,10). However, in some regions of the world the prevalence of pediatric urolithiasis has significantly increased in a way that it is comparable to that of adults (11).

Reliable reports from different countries show that the prevalence of urolithiasis is increasing persistently (9,10,12). Over the past three decades, the prevalence rate of renal stone in the United States has doubled. Similar data from Southeast Asia and most European countries with increase in the prevalence of stone disease can be found in the literature (13). This increasing prevalence of renal stone in children is age dependent and can be attributed to the corresponding rise in diabetes, obesity, and hypertension especially in older age groups (14). In the first decade of life, it is more prevalent in boys while in the second decade, it is female-predominant (15). Increased awareness of the issue and extensive use of ultrasonography in routine practice for children with different urinary symptoms may explain the increasing prevalence of renal stone disease. Modern ultrasonographic equipments in recent years and increased experience has enabled sonologists to detect renal deposits of less than 3 mm in size (5,16).

The incidence of urolithiasis is increasing all around the world (13). Changes in the socio-economic status of the people in different countries might be the main cause of changes in the incidence and type of renal stone (17). Also an increase in using antibiotics by children has been considered as a possible risk factor for increased incidence of pediatric renal stone (18).

The overall incidence of nephrolithiasis in childhood is estimated to be about 10% of the reported rate in adults. This incidence is estimated to be about 5% in children living in industrialized countries (19). The explanation for the lower incidence of renal stone in pediatric age group is not exactly known. One possible explanation for the lower incidence may be the higher urinary concentrations of some inhibitors of crystal formation like magnesium and citrate in pediatric age group as compared to adults (20,21).

On the other hand, the reported incidence numbers should be considered with caution because a significant number of the patients with nephrolithiasis may be asymptomatic and remain undetected with an incidental detection rate of 15%–40% (22). Taking into account the difficulties in ultrasonographic diagnosis of RM, the difference between reported and real incidence of RM is probably even more significant than that of renal stone.

## Clinical significance

Renal stone can be considered as a chronic disease with a recurrence rate of more than 50% during ten years of follow-up (23). RM is a separate entity which is more frequent in younger children and especially in infants (6).

Despite great investigations during the past few decades for better understanding of the etiology, physiopathology, management and prevention of renal stone in children, clinical importance and natural course of RM remain unclear. RM can be detected in first few months of life and even in the neonatal period and might be the source of late childhood renal stone (24). Although it seems to be mainly the disease of infancy, RM can be detected in all pediatric and adult age groups (25-28).

RM has been observed mostly in children with abdominal pain, urinary tract infection and urinary symptoms such as hematuria and dysuria either isolated or in combination (5). RM possibly represents the first step in calculus formation and it has usually been considered that there could be an association between RM and urinary stone at older ages (28).

## Etiology

Majority of pediatric patients with RM have one or more underlying metabolic disorders similar to those with renal stone (6). Urinary tract infection is a significant cause or complication of renal stone in children; however, its role in RM is not so clear. Elevated serum levels of vitamin D may have a significant role in infants with stone formation and RM (29). Different anatomical anomalies of the kidneys and urinary tract can predispose to urolithiasis: in a large series of 511 pediatric patients with urolithiasis, 12% had anatomical abnormalities (30).

## Pathogenesis

Different theories for pathogenesis of renal stone and RM imply that their mechanism of formation is too complicated. Formation of calcium oxalate stone, as the most common form, has a multistep pathogenesis including nucleation, crystal growth, crystal aggregation, and finally crystal retention. Currently, renal stone formation mechanism has many unclear aspects. With better understanding of this complex mechanism, it might be possible to develop a new successful strategy to prevent stone formation (31).

## Clinical manifestations

The presenting symptoms of RM in children are mainly similar to renal stone (6,7,28). Therefore, RM and urolithiasis cannot usually be differentiated according to the clinical manifestations (32). Renal stone or RM may be asymptomatic (9). Spontaneous stone passing can occur in all ages (9,33). In infants, symptoms of renal stone or RM may be confused with infantile colic (9). Among symptomatic children with renal stone, the most common presentations are abdominal or flank pain, dysuria, vomiting, oliguria, hematuria, sterile pyuria and urinary tract infection (4,9,30). Macroscopic or microscopic hematuria may be detected in up to 90% of children with urolithiasis (34). Positive family history is observed in most children with renal stone (4,9).

## Diagnosis

Systematic diagnostic evaluation in children with suspected renal stone is usually started with a history, followed by careful physical examination and imaging studies, usually not including plain x-ray as an initial evaluation (9). Ultrasonography is suggested as the primary imaging modality for diagnosis of suspected pediatric urolithiasis (35,36). Most of the reported cases of RM in children have been diagnosed by ultrasonography (5,37). When the result of this examination is uncertain, unenhanced helical computed tomography (CT) scan will most probably add further proper information and contribute to the definite diagnosis (35,36).

Conventional radiography (kidney-ureter-bladder view) may not detect small even radio-opaque stones in the kidney or ureter and yields no information about possible obstruction. Therefore, it is not good enough for diagnosis of RM. Diagnostic yield of intravenous pyelography is much less than non-contrast CT scan and it exposes the patient to the risk of radiocontrast agents (38).

Helical CT scan without contrast is the imaging of choice in patients with suspected RM or nephrolithiasis. It has several benefits comparing to other imaging techniques: it needs no radiocontrast material, shows the stone regardless of location even in the distal ureter and can detect both radio-opaque and radiolucent stones even as tiny as 1-2 mm in diameter. Furthermore, hydronephrosis and abdominal or renal disorders apart from renal stone that might be the cause of the patient’s symptoms can be detected with helical CT scan (38,39). Helical CT scan, as the gold standard technique in renal stone diagnosis, has been reported to have the overall accuracy of 98% (40). The main exception to this rule for helical CT scan is in the diagnosis of indinavir stone which can form in patients being treated for the human immunodeficiency virus (41).

Appearance of symptoms suggestive of urolithiasis in spite of normal ultrasound and plain x-ray film is near nine times higher than developing overt renal stone in children with urinary solute abnormalities that can predispose to renal stone. This difference might be due to several factors like limited sensitivity of the aforementioned methods of imaging or crystaluria that can damage urinary epithelium before stone formation. Moreover, imaging evaluations performed with a short interval after the passage of stone will give negative results (42). Therefore, repeated periodic evaluations may be indicated in patients with recurrent unexplained abdominal pain, dysuria, and/or hematuria with or without associated hypercalciuria (7,37).

## Metabolic evaluation

Metabolic abnormalities can be found in majority of pediatric patients with renal stone and RM (5,43,44). Due to the high risk of recurrences in childhood nephrolithiasis and RM, especially if associated with treatable conditions such as idiopathic hypercalciuria, hypocitraturia, renal tubular acidosis and cystinuria, a complete metabolic evaluation is indicated in all children with nephrolithiasis and RM (3,45).

Hypercalciuria and hypocitraturia are the most commonly reported metabolic abnormalities in patients with renal stone and composition of majority of the childhood renal calculi is either calcium oxalate or calcium phosphate (3,46). In a study on 153 infants and children with renal stone in our center, 90.8% had at least one metabolic abnormality with hypomagnesiuria and hypocitraturia as the most common findings (47).

## Treatment

Spontaneous renal stone passing may occur in 51.21% of the affected children and it is expected to be even higher in children with RM. Follow-up conservative therapy, consisting of high fluid intake, dietary manipulations and addition of potassium citrate and/or chlorothiazide in high risk children, is reported to be sufficient for prevention of stone recurrence in 92.1% of the patients (48). Dietary manipulation is not feasible during the first 6 month of life, especially in those with exclusive breast feeding.

Since dietary habits play a crucial role in the pathogenesis of renal stone, physicians’ recommendation of proper nutrition and fluid intake is of great importance. Increased fluid intake and a low salt diet (2-3 mEq/kg/day) is recommended in all children with any type of stone disease (3,17). Considering the underlying cause of renal stone or RM, additional drugs or other non-pharmaceutical treatment may be indicated.

Long-term non-pharmaceutical medical treatment is indicated in all children with their first documented renal stone or RM. Pharmaceutical therapy is suggested for those with initial multiple stones, recurrences during a short interval or presence of underlying metabolic disorders. Pharmacotherapy is also indicated for children in whom fluid and dietary therapy is ineffective in controlling the stone disease and for those with primary hyperoxaluria, renal tubular acidosis, cystinuria, hypomagnesuria, hypocitraturia or idiopathic hypercalciuria (3). Treatment with either potassium citrate (2-4 mEq/kg/day) or magnesium citrate or their combination is effective in most patients with stone disease, with or without underlying metabolic abnormalities (49,50). Sodium citrate is mostly thought to be less suitable because it can increase sodium delivery to the nephron and augment the risk of stone formation (3,50).

Regarding the drugs used for pain control in renal colic, randomized controlled trials suggest that parenteral nonsteroidal anti-inflammatory drugs are as effective as narcotics (51). Taking into account the small size of RM and therefore minimal risk of urinary tract obstruction, urological intervention is not usually indicated.

## Prevention

Long term conservative therapy, consisting of dietary manipulations, adequate fluid intake and in special conditions appropriate drug therapy, is effective for prevention of stone recurrence in most of the patients (48). Fruits and vegetables are useful to prevent calcium oxalate, cystine and uric acid stones because of increasing the urine pH. However, in struvite or phosphate containing stones, cranberry juice or betaine is recommended to decrease urine pH (14).

## Prognosis

Randomized controlled trials suggest that the passage rate for ureteral calculi is surprisingly not different in different age groups; thus, in all ages, stones greater than 5 mm are rarely passed spontaneously (52). In some reports, spontaneous stone passage in children is even more common as compared to adults (1).

The children with one or more metabolic abnormalities are about fivefold more likely to develop recurrent stones than those with normal metabolic evaluations (5,52). Therefore, it is important to evaluate all children with renal stone including those with RM for metabolic abnormalities to identify those with underlying metabolic disorders (52).

There are only few reports in the literature about long-term prognosis of RM and we found no randomized controlled study on the management and outcome of this common entity. To our knowledge, only about 257 cases with RM have had more than six months of follow-up (5,7). In a report on 292 children with RM, mean follow-up was 14.6 ± 5.9 months for 228 cases (5). During follow-up of these 228 patients with RM, about 1/3 progressed, 1/3 did not change or decreased and 1/3 disappeared (5). In another report on 196 pediatric patients with RM, only 29 patients had follow-up of two years or more. In the latter study, nine patients developed 4-7 millimeters stones later on (7).

## Conclusion and recommendation

RM is a disorder with increasing prevalence in smaller children. It has metabolic risk factors and clinical manifestations similar to urolithiasis and can lead to renal stone further along. Helical CT scan without contrast is the imaging of choice in patients with suspected RM. RM is expected to have spontaneous passage in most of the affected children and follow-up conservative therapy without urologic intervention is probably sufficient to avoid its recurrence in most of the patients. However, further longitudinal investigations are needed to be conducted into clinical approach and natural course of this disorder.

## Authors’ contribution

All authors contributed to the study. MAF, JH and MHF conducted the search and prepared the primary draft. All authors read and approved the final manuscript.

## Conflicts of interest

The authors declared no competing interests.

## Ethical considerations

Ethical issues (including plagiarism, data fabrication, double publication) have been completely observed by the authors.

## Funding/Support

None.
